# Self-Esteem Among the Elderly Visiting the Healthcare Centers in Kermanshah-Iran (2012)

**DOI:** 10.5539/gjhs.v7n5p352

**Published:** 2015-04-15

**Authors:** Jafari Franak, Khatony Alireza, Mehrdad Malek

**Affiliations:** 1 Kermanshah School of Nursing and Midwifery, Kermanshah University of Medical Sciences, Kermanshah, Iran; 2 Social Development and Health Promotion Research Center, Kermanshah University of Medical Sciences, Kermanshah, Iran

**Keywords:** self-esteem, elderly, counseling centers, psychological development

## Abstract

**Background and Objective:**

Self-esteem is viewed the most decisive factor in the psychological development of the elderly. This study was performed to assess self-esteem among the elderly referring to the elderly consulting unit of the healthcare centers in Kermanshah, Iran.

**Methods:**

A cross-sectional study was completed with 201 elderly respondents visiting the consulting unit of the healthcare services in Kermanshah, Iran. The samples were selected through convenience sampling. Rosenberg Self-esteem Scale (RSC) was used to gather the required data. Data were analyzed by using both descriptive (frequency, mean, median and standard deviation) and inferential statistics (chi-square and independent t-test).

**Results:**

The findings showed a mean of 35.63±5.25 for self-esteem, indicating a high level of self-esteem (66.2%) among the elderly. A statistically significant difference was reported between the mean of self-esteem and career (p<0.001), marital status (p<0.04), history of health problems (p<0.04), residence (p<0.001), education (p<0.001) and income (p<0.001).

**Conclusion:**

The findings of this study indicated that approximately one third of the elderly had a low self-esteem, which is indicative of the need to promote the self-esteem of the elderly in order to reduce their physical, psychological and social problems. Thus, it is necessary for the healthcare authorities to provide the elderly with financial, social and psychological support.

## 1. Introduction

Ageing is a critical and crucial stage of human development which is, contrary to the common belief, not only the end of life but also a natural process of life (Pourjafar et al., 2010). Ageing is the outcome of the natural course of time that leads to physiological, mental and social changes and is not limited to a specific group, and everybody will gradually experience it (Shahbazzadegan et al., 2008). Disability is a common problem among the elderly which leads to reduction of patience in dealing with unwanted stimuli, lowered performance in unfavorable environmental conditions, depression, increase of stress, lack of compatibility in coping with responses and reduction of self-esteem or even death.

Self-esteem is the belief in one’s ability to think, confidence in one’s right for achievement, happiness and worthiness, and expression of the needs and desires (Shahbazzadegan et al., 2009). Self-esteem is associated with the beliefs and images we have about ourselves, and is a measurement of how much we love and accept ourselves or others (Navabinejad, 2008). Self-esteem is closely associated with a person’s mental image about oneself as well as the coping style. The results of Nokani et al. (2006) indicated that a positive image about one’s body creates a sense of worthiness in the person, and conversely, the mental image that is undergone a change leads to changes in the sense of worthiness (Nokani et al., 2006).

There are various views about self-esteem and its effects on social and psychological development, among which Ericson’s view about psychosocial development, owing to strong theoretical grounds, has been attracted by many researchers (Ochse & Plug, 1986; Slater, 2003). Ericson believes that psychological development depends on specific social relations that the individual creates during different stages of development. He also argues that the final stage of development is in the ageing period. The more a person has efficiently coped with problems in the past, the more he/she experiences a sense of development and perfection. The antithesis of perfection is disappointment. An aged person who is unpleased with the past events of life feels hopeless and desperate and views the life and social relations with spite and hatred (Abdoli et al., 2010). In this regard, Mahdavi et al. (2008) concluded that the more the people enjoy self-confidence and self-esteem, the more efficient they will become and be protected from psychosocial problems.

Various domestic and international studies have investigated self-esteem and the factors associated with it in the elderly. Orth et al. (2010) reported that the increase of self-esteem in all age groups, especially the elderly is directly related to optimism, positive affect and a sense of social support, and is inversely correlated with negative affect. Park et al. (2014) found out that the elderly with health behaviors such as physical activity are more probable to successfully spend their ageing period. In line with these views, a primary objective of caring for the elderly is helping them to preserve maximum independence in a safe environment to promote their life quality and to minimize the healthcare costs by preventing physical injuries (Safavi Bayat & Zoori Styne, 2008; Khalili et al., 2012). Since the elderly with high self-esteem can more easily cope with the threats and stressful events of life without negative experience and psychological disorders, and due to lack of information about the level of self-esteem in the elderly visiting the counseling centers of Kermanshah healthcare services, the present study was carried out to explore this growing area of concern.

## 2. Methods

In this cross-sectional study conducted in Kermanshah healthcare services in 2012, all the elderly visiting the elderly counseling center in the first three-month of the year were selected through convenience sampling. The criteria for inclusion in the study were minimum age of 61 years, having a healthcare file in the elderly counseling centers and willingness for participation in the study.

A two-section questionnaire was used to collect the data. The first section of the questionnaire involved demographic information, including age, gender, marital status, economic status, career, education, history of health problems and residence. The second section was the Rosenberg Self-esteem Scale (RSC), whose validity and reliability have been confirmed by different domestic and international studies. Tafarodi and Sawan reported the reliability index of 0.80 for this scale using test-retest method (Tafarodi & Sawan, 2001). Also, Kendlerd, Myers and Neale reported the reliability index of 0.89 for this scale through internal consistency measure (Masoudnia, 2010). Further, Muhammadi (2005) reported Cronbach’s alpha coefficient and split-half index of 69% and 68% for this questionnaire, respectively. He also reported the test-retest reliability indices of 77%, 73% and 78% for this scale over one week, two weeks and three weeks intervals, respectively.

The Rosenberg Self-esteem Scale is one of the most common scales for self-esteem measurement which provides an overview of the positive and negative attitudes about self. Some of the items of this scale include: “I have positive attitude about myself”, “I am generally satisfied with myself”, I sometimes feel I am useless”, I sometimes think I cannot do anything” and “I feel I have some good characteristics” (Shahbazzadegan, 2008).

The Rosenberg Self-esteem Scale used in this study comprised of 10 items based on 5-point Likert scale, including strongly agree, agree, undecided, disagree and strongly disagree. The samples selected one option for each item as the criterion compatible with them. The choices were scored from 1 to 5 (5=strongly agree, 4=agree, 3=undecided, 2=disagree and 1=strongly disagree). The total range of scores was 10-50 and the cutoff point was considered to be 34. The samples were categorized in low self-esteem (≤34) and high self-esteem (≥34) groups according to their score (Masoudnia, 2010).

To collect the required data, after obtaining permission from the vice chancellery of research and technology of the university, the researcher visited the counseling centers of Kermanshah healthcare services and initiated sampling. To this end, the objectives of the study were explained to the respondents, the confidentiality of the demographic information and responses were assured, and informed consent for participation in the study was taken from the respondents. The questionnaires were completed by the respondents and were collected by the researcher. To analyze the collected data, descriptive statistics (frequency (%), mean and standard deviation) and inferential statistics (one-way ANOVA and independent t-test) were applied. Independent t-test was used to compare the self-esteem score in terms of two-state qualitative variables (gender, residence, history of health problems) and one-way ANOVA was used for multiple qualitative variables (career, education, marital status) and ranking quantitative variables (age and economic status). The statistical significance level was set at p<0.05.

## 3. Results

A total number of 201 elderly participated in this study, from whom 108 (53.7%) were female and 154 (76.6%) were married. 107 (53.2%) of the samples were in the age range of 66-75 with the mean age of 68.5±27. Seventy (34.8%) respondents were less educated, 90 (44.8%) were homemakers, and 77 (38.3%) had good economic status. One hundred and sixty four (81.6%) of the participants owned a personal dwelling (Table 1). One hundred and fifty three (76.1%) participants had a history of health problems, including hypertension (n=58, 26.12%), digestive problems (n=28, 12.61%) and arthritis (n=25, 11.26%). The mean of self-esteem in the elderly was 35.63±5.25. The maximum and minimum scores for self-esteem were 20 and 46, respectively. Seventy five (33.8%) elderly had low self-esteem and 146 (66.2%) enjoyed high self-esteem. The maximum level of self-esteem in the elderly was reported for academic education (100%) and the minimum level was reported for unemployment (10%). The highest means of self-esteem were reported for males (39.3), married samples (35.97), samples owning a private dwelling (36.27), participants with academic education (38.68), retired samples (36.5) and samples with no history of health problems (37.95).

**Table 1:** Demographic characteristics of samples in terms of mean self-esteem

Variables	Group	No(%)	Mean±Sd	*p.v*
Age	61-65	55(27.4)	35.21±5.21	**NS**
	66-75	107(53.2)	35.8±5.05	
	76≤	39(19.4)	35.9±5.9	
Gender	male	93(46.3)	39.3±5.2	**NS**
	female	108(53.7)	35±5.3	
Marital status	single	3(1.5)	29.66±10	**0.04**
	married	154(76.6)	35.97±5.1	
	divorced	5(2.5)	31.2±5.7	
	widowed	39(19.4)	35.3±5	
Residence	Personal	164(81.6)	36.27±5.6	**<0.001**
	Rental	37(18.4)	32.27±4.9	
Education	Illiterate	70(34.8)	33.25±5.6	**<0.001**
	Elementary	13(6.5)	37±8.1	
	Middle school	62(30.8)	36.2±4.3	
	High school	31(15.4)	36.77±4	
	University	24(12.4)	38.68±5.2	
Career	Retired	82(40.8)	37.1±5.2	**<0.001**
	Self-employed	10(5)	36.5±4.8	
	Homemaker	90(44.8)	35.25±5	
	Worker	9(4.5)	30.87±2.7	
	Unemployed	10(5)	29.8±6.3	
History of health problems	yes	153(76.1)	31.12±5.7	**0.043**
	No	8(23.9)	34.9±5.1	
Economic status	Good	77(38.3)	38.14±3.7	**<0.001**
	Moderate	73(36.3)	35.06±5.96	
	Week	51(25.4)	32.62±5.25	

The results of the comparison of the means for self-esteem indicated a significant difference in terms of marital status (p=0.04); the married and single samples obtained the maximum and minimum levels of self-esteem, respectively. Moreover, the comparison of the means of self-esteem in the elderly showed a significant difference for education variable (p<0.001); the maximum and minimum levels of self-esteem were reported for the samples with academic education and illiterate samples, respectively.

The comparison of the means also revealed a significant difference between self-esteem for career variable (p<0.001); the retired and self-employed samples obtained the maximum and minimum levels of self-esteem, respectively. The findings also showed that the mean of self-esteem in the elderly owning a private dwelling was significantly higher than the samples living in rental dwellings (p<0.001).

In addition, the results indicated a significant difference between the means of elderly self-esteem and the economic status (p<0.001) so that the elderly with good economic status obtained the maximum self-esteem and those with poor economic status gained the minimum levels of self-esteem. Regarding the history of health problems, a significant difference was observed in the means of self-esteem (p=0.043); the elderly with no history of health problems had a higher self-esteem than the elderly with a history of health problems. However, no significant difference was reported for the elderly self-esteem in terms of age and gender (Table 1).

## 4. Discussion

The current research was carried out to analyze self-esteem among the elderly visiting Kermanshah healthcare services. The findings of the study showed a generally high level of self-esteem in the elderly. In a study performed in Korea, the mean of self-esteem was reported to be 28.23 (Chui et al., 2011). In another study carried out in Ecuador, the mean of self-esteem in the middle-aged women was 26.6±3.1, which is lower than the mean obtained in the present study (Chedraui et al., 2010). This difference can be due to the cultural differences in various countries. It should be noted that, owing to dominant traditional culture in the Iranian community, the elderly are highly respected, which can positively affect their self-esteem.

Further, the results showed similar levels of self-esteem in different age groups. Pruessner et al. (2004) argued that self-esteem is a component that is independent of age. However, McMullin & Cairney (2004) suggested that older age groups have lower levels of self-esteem due to changes in their physical health status, which consequently causes negative changes in self-perception.

The difference between the results of these studies and the present study can be due to cultural differences.

Also, the findings indicated no significant difference between males and females in terms of self-esteem. The results of the current study are in line with the findings of Noghani et al. (2006), but McMullin and Cairney (2004) believe that women have lower self-esteem than men. Moreover, Hong et al. (1993) suggested that males have higher levels of self-esteem than females; however, some studies have shown higher levels of self-esteem for women than men (Richardson & Benbow, 1990; Mirzaeyalvijeh, 2012). Lower levels of self-esteem in women than men may be indicative of the fact that females undergo higher levels of depression and stress (Brown et al., 1990). The authors of the present study tend to think that the difference between the results of the current study and other studies can be due to the cultural differences in various regions.

In addition, the results of this study showed maximum and minimum levels of self-esteem for the married and single elderly. Brown & Bifulco (1990) reported a significant difference between self-esteem and marital status and Jang (2006) reported a low level of self-esteem for the people who lived alone.

Martinez-Vallarreal et al. (2007) found out that the elderly women who live alone have lower self-esteem. However, Fathi Ashtiani et al. (2008) reported lack of relationship between marital status and self-esteem. The authors believe that higher levels of self-esteem in the married people may be associated with the support they receive from their partners; something the divorced people are deprived of, which consequently lead to lower self-esteem in them.

Furthermore, the obtained findings indicated higher self-esteem for the elderly owning private dwellings than the elderly living in rental dwellings. The results of the study by Antonelly (2000) showed higher level of self-esteem for the elderly living in private dwellings than those living in nursing homes. Also, Rezaei and Manouchehri (2009) stated that the elderly living in nursing homes have poorer mental and emotional conditions than those living in private dwellings. Dwelling is an important physiologic need of every person. Owning a private dwelling and a sense of ownership remarkably creates feelings of satisfaction and higher self-esteem in the elderly.

Additionally, the findings showed higher level of self-esteem in the elderly with academic education than those with no education. The results of a study showed a higher level of self-esteem in the people with academic education (Nosek et al., 2003). Miroesky and Rose (1996) argued that people with academic education had a higher level of self-esteem than less educated people. Also, Rosenberg and Pearlin (1978) reported that the elderly with poor education have lower self-esteem. The authors tend to think that education is one of the factors that can help the elderly to cope with the changes caused by the ageing process.

In the present study, the retired elderly had higher self-esteem than the unemployed elderly. Rosenberg and Pearlin (1978) reported that the elderly with low-income careers have a lower level of self-esteem. The authors of the current survey believe that career and income affect the amount of self-esteem. Since the financial needs of the elderly increase because of medical services and family expansion, their income should necessarily increase consequently; this is not so, however, in most of the occasions. Although the pension of the retired elderly has increased in recent years, it is not sufficient to satisfy their financial requirements, thereby influencing their self-esteem.

The findings also indicated higher self-esteem for the elderly with no history of health problems than those with a history of health problems. The results of the study conducted by McMullin and Cairney (2004) showed that the people with poor health have lower self-esteem. Further, the findings of Nosek et al. (2003) revealed that health problems reduce self-esteem. Physical disorders expose humans to many obstacles and limitations. Physical ailments are such problems that in most cases cause disability and despair in the elderly. Due to the age-related physiological changes in the elderly, these disabilities are increased, which in turn may increase mental problems and low self-esteem in them. Hence, low levels of self-esteem in the elderly may probably be associated with physical problems.

Finally, the results of this study indicated that the elderly with good economic status have higher self-esteem than those with poor economic status. In line with this, Shahbaz Zadegan et al. (2008) reported low levels of self-esteem for people with low income. However, Fathi Ashtiani et al. (2008) concluded that monthly income has no effect on the elderly’s self-esteem. The authors of present research tend to think that the elderly with low income have problems in fulfilling the basic needs of their families, which consequently creates preoccupations, thereby affecting their self-esteem.

One of the limitations of the current study was poor generalizability of the findings due to individual, cultural and social characteristics of the samples. On the other hand, data were collected through self-report, which may have affected the accuracy of the results. Another limitation of this study was the sampling technique, which was carried out by convenience sampling method.

This study was conducted on the elderly referring to the counseling centers of healthcare services. Further studies are suggested to explore the self-esteem of elderly in nursing homes or in private clinics.

## 5. Conclusion

The findings showed that most of the elderly enjoyed high levels of self-esteem, although one third of the samples had poor self-esteem. The single, low-income, less educated and unemployed elderly as well as those with a history of health problems and those living in rental dwellings had low levels of self-esteem. The results of the comparison of the self-esteem means indicated a statistically significant difference for career, marital status, and history of health problems, residence, education and income. Given the significance role of self-esteem in reducing physical, psychological and social problems of the elderly, it is necessary for the healthcare authorities to provide the elderly with financial, social and psychological support.
